# Supplementary material for: Dual Roles of Plasma miRNAs in Myocardial Injuries After Polytrauma: miR-122-5p and miR-885-5p Reflect Inflammatory Response, While miR-499a-5p and miR-194-5p Contribute to Cardiomyocyte Damage
Source: Cells. 2025 Feb 18;14(4):300. doi: 10.3390/cells14040300 (PMC11854772; doi:10.3390/cells14040300)
Supplement: Supplementary file 1 [file cells-14-00300-s001.zip › cells-3455144-supplementary.pdf]

Supplementary data

Gene Expression Analysis

For gene expression analysis, cells were washed as described before, lysed with RLT lysis buffer (Qiagen, Hilden, Germany) and total RNA was extracted using the RNeasy Mini Kit (Qiagen, Hilden, Germany), according to the manufacturer's protocol. RNA concentration and purity were assessed using a plate reader (Tecan Infinite M Plex, Crailsheim, Germany). cDNA was synthesized from DNase-treated RNA using iScript cDNA synthesis kit (Bio-Rad, Puchheim, Germany). Quantification of gene expression was performed via RT-qPCR, using 3 µL cDNA per reaction with iTaq Universal SYBR Green Supermix (Bio-Rad, Puchheim, Germany). Amplification was conducted on a CFX96 Touch Real-Time PCR Detection System (BioRad, Puchheim, Germany). Commercial human gene-specific primers including IL-1B, TLR2, NLRP3, and GAPDH were purchased from Qiagen (Qiagen, Hilden, Germany). GAPDH served as the endogenous control for normalization, and relative gene expression was calculated using the delta-delta Ct method ($2^{-\Delta\Delta Ct}$). All samples were run in duplicate, with three technical replicates for each sample.

Supplementary Table S1. Demographic and clinical features of polytrauma patients and healthy controls. TnT: Troponin T; PT: Polytrauma; ISS: injury severity score; n/a: not applicable

	**Cohort 1**			**Cohort 2**			**Cohort 3**		
	TnT High (n = 5)	TnT Low (n = 5)	P value	TnT High (n = 10)	TnT Low (n = 9)	P value	PT (n = 15)	Healthy (n = 7)	P value
**Age (years)**	57 ± 6	38 ± 17	0.23	48 ± 12	43 ± 17	0.50	55 ± 22	43 ± 14	0.21
**Gender (Male/Female)**	5/0	4/1	0.35	9/1	7/2	0.50	11/4	5/2	0.93
**TnT (pg/mL)**	565 ± 379	3 ± 3	0.01	478 ± 297	4 ± 5	0.00	24 ± 25	n/a	
**ISS**	30 ± 16	39 ± 7	0.29	34 ± 17	38 ± 7	0.53	28 ± 10	n/a	
**creatinine** (mg/dL)	1.09 ± 0.20	1.04 ± 0.19	0.75	1.12 ± 0.31	0.97 ± 0.26	0.29	1.02 ± 0.24	n/a	

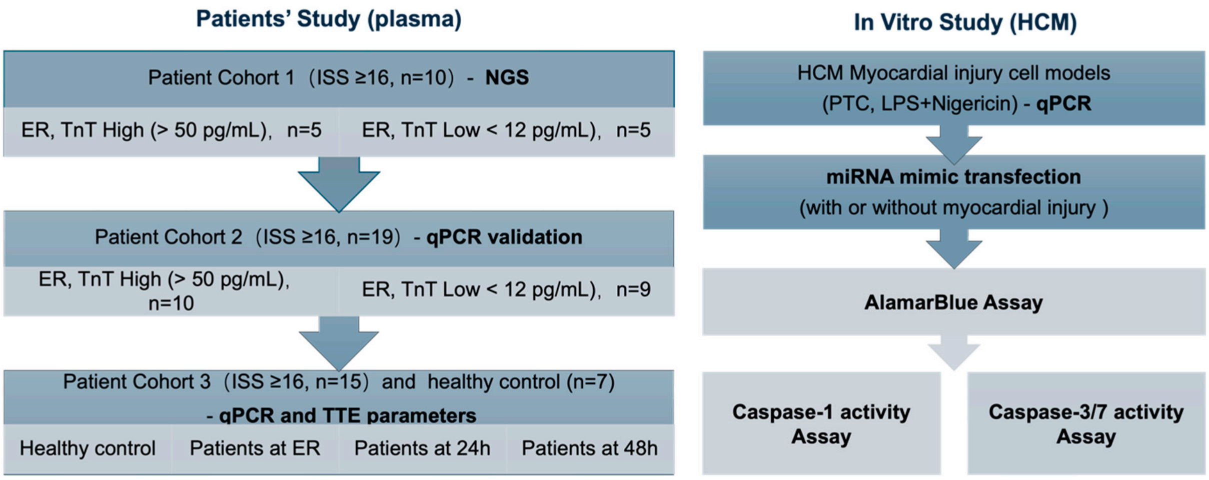

Supplementary Figure S1. Graphical summary of the study design. NGS: next generation sequencing; ISS: injury severity score; TnT: Troponin T; TTE: Transthoracic echocardiography; HCM: human cardiomyocyte.

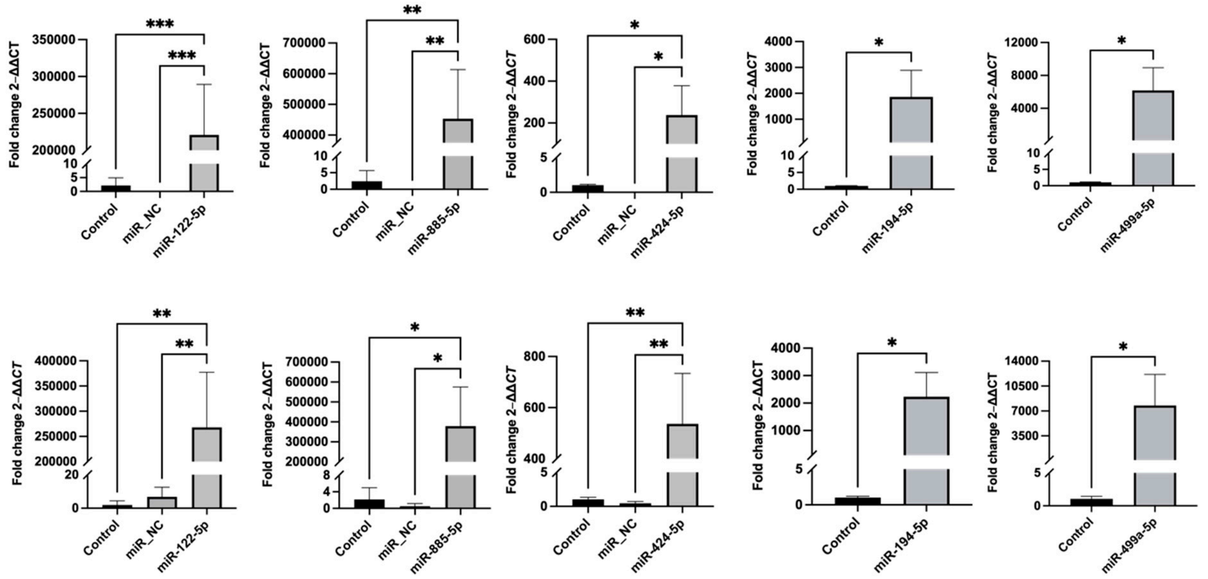

Supplementary Figure S2. Transfection efficiency of miRNA mimic was confirmed using qPCR. (A) Relative quantification of miRNA expression after miRNA mimic transfection was performed using the delta Ct method ($2^{-\Delta\Delta Ct}$) with normalization to the spike-in control cel-miR-39-3p. (B) Relative quantification of miRNA expression after miRNA mimic transfection was performed using the delta-delta Ct method ($2^{-\Delta\Delta Ct}$) with normalization to the spike-in control UniSp6. $^{*}p < 0.05$, $^{**}p < 0.01$, $^{***}p < 0.001$. miR_NC: miRNA mimic negative control.

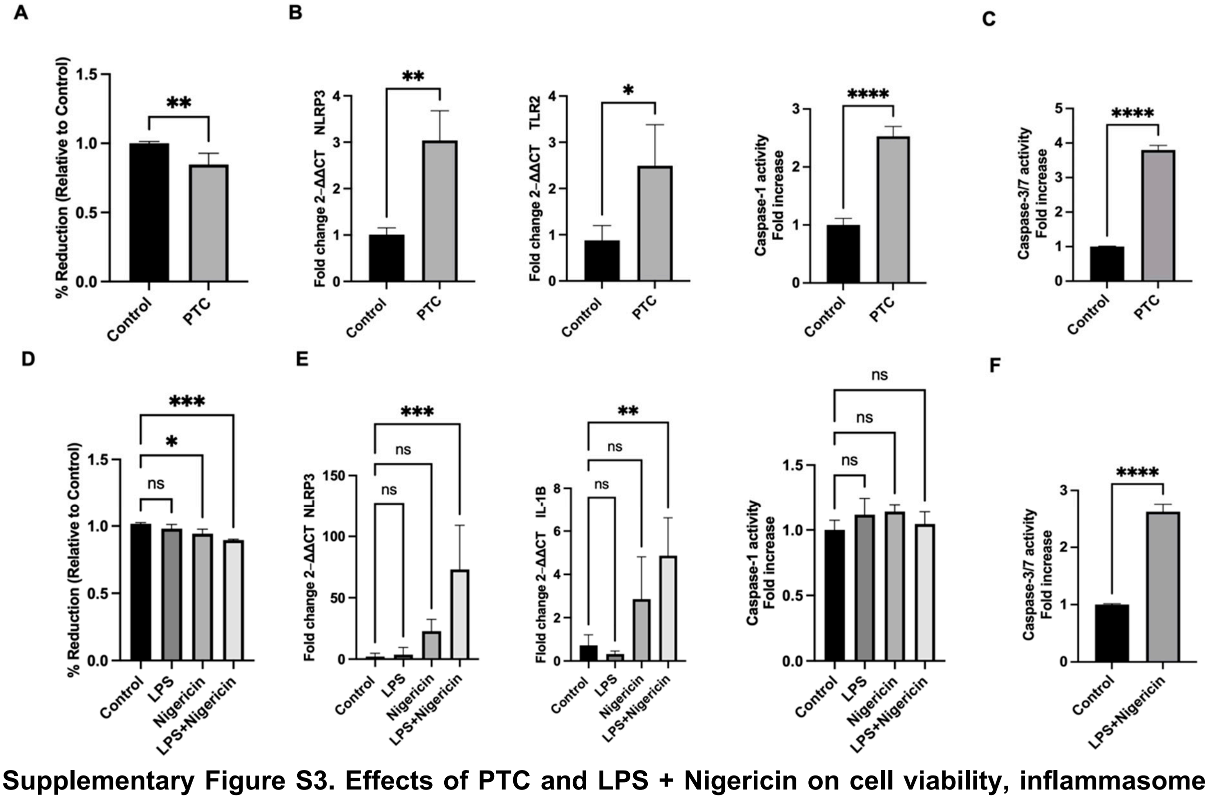

Supplementary Figure S3. Effects of PTC and LPS + Nigericin on cell viability, inflammasome activation, and apoptosis in human cardiomyocytes (HCM) cells. (A) HCM cells were treated with PTC for 4 hours. Cell viability was evaluated using the alamarBlue assay. (B) HCM cells were treated with PTC for 4 hours, followed by qPCR analysis to assess the expression of inflammasome-related genes, NLRP3 and TLR2. Extracellular caspase-1 activity was then measured using the luminescent

Caspase-Glo®1 inflammasome assay. (C) HCM cells were treated with PTC for 4 hours, and apoptosis was quantified by measuring the Caspase-3/7 activity assay. (D) HCM cells were stimulated under the following condition: LPS stimulation for 4 hours, Nigericin stimulation for 1 hour in serum-free condition, or LPS stimulation for 4 hours followed by Nigericin stimulation for 1 hour in serum-free condition (LPS + Nigericin). Cell viability was then measured using the alamarBlue assay. (E) Gene expression of inflammasome-related genes, NLRP3 and IL1B, was determined using qPCR following LPS stimulation for 4 hours, Nigericin stimulation for 1 hour, or LPS + Nigericin stimulation. Extracellular caspase-1 activity was measured using the luminescent Caspase-Glo®1 inflammasome assay. (F) Caspase-3/7 activity was analyzed to quantify apoptosis following LPS + Nigericin stimulation. *$p < 0.05$, **$p < 0.01$, ***$p < 0.001$, ****$p < 0.0001$. NLRP3: NOD-like receptor protein 3; TLR2: Toll-like receptor 2; IL-1B: interleukin-1 beta.
